# The Direct and Indirect Costs of Colorectal Cancer in Vietnam: An Economic Analysis from a Social Perspective

**DOI:** 10.3390/ijerph18010012

**Published:** 2020-12-22

**Authors:** Binh Thang Tran, Kui Son Choi, Thanh Xuan Nguyen, Dae Kyung Sohn, Sun-Young Kim, Jae Kyung Suh, Van Sang Phan, Huu Tri Pham, Minh Hanh Nguyen, Thanh Binh Nguyen, Huu Khoi Hoang, Thi Thanh Binh Nguyen, Minh Tu Nguyen, Jin-Kyoung Oh

**Affiliations:** 1 Department of Cancer Control and Population Health, Graduate School of Cancer Science and Policy, National Cancer Center, Goyang 10408, Korea; tranbinhthang@hueuni.edu.vn (B.T.T.); kschoi@ncc.re.kr (K.S.C.); gsgsbal@ncc.re.kr (D.K.S.); sykim@ncc.re.kr (S.-Y.K.); 2 Faculty of Public Health, Hue University of Medicine and Pharmacy, Hue University, Hue City 52000, Vietnam; phanvansang94@gmail.com; 3 National Cancer Control Institute, National Cancer Center, Goyang 10408, Korea; 4 Hue Central Hospital, Hue City 52000, Vietnam; thanhxuanbvh@gmail.com (T.X.N.); phamhuutri05@gmail.com (H.T.P.); minhhanhyk1016@gmail.com (M.H.N.); 5 Center for Colorectal Cancer, Research Institute and Hospital, National Cancer Center, Goyang 10408, Korea; 6 National Evidence-Based Healthcare Collaborating Agency, Seoul 04554, Korea; jksuh81@gmail.com; 7 Family Hospital, Da Nang City 550000, Vietnam; Drbinhnguyenno1@gmail.com; 8 Faculty of Medicine, Da Nang University of Medical Technology and Pharmacy, Da Nang City 550000, Vietnam; khoimat@gmail.com; 9 Department of Pediatrics, Hue University of Medicine and Pharmacy, Hue University, Hue City 52000, Vietnam; nttbinh.med@hueuni.edu.vn; 10 Undergraduate Training Office, Hue University of Medicine and Pharmacy, Hue University, Hue City 52000, Vietnam; nmtu@huemed-univ.edu.vn

**Keywords:** health care costs, economics burden, colorectal neoplasms, Vietnam

## Abstract

The incidence and mortality of colorectal cancer (CRC) has increased rapidly in Vietnam, but the economic burden of this disease has never been estimated. We estimate the direct and indirect cost of CRC patients in Vietnam in 2018 using a prevalence-based approach and human capital method. The total economic cost of CRC was VND 3041.88 billion (~$132.9 million), representing 0.055% of the 2018 gross domestic product. Notably, indirect costs comprised 83.58 % of the total cost, 82.61% of which is future income loss, because CRC occurs during productive years. The economic burden of CRC in Vietnam is substantial. The medical cost for CRC diagnosis and treatment is higher for younger patients and for those in advanced stages. Strategies to decrease the economic burden of CRC at the patient and national level, such as screening programs, should be developed and implemented in Vietnam.

## 1. Introduction

Globally, the incidence of colorectal cancer (CRC) is high and positively associated with economic development. However, the prevalence trend of CRC has been stabilizing or decreasing in high-income countries, while it has been rising continuously in low-income and low-middle income countries [1,2]. These different patterns could be explained by the early diagnosis and treatment in wealthy countries, whereas a large portion of late-stage diagnosis was observed in poor countries [1,2].

In Vietnam, CRC is one of the five most common cancers, with an age-standardized incidence of 21 per 100,000 inhabitants in 2015. This shows a remarkable increase in incidence compared with previous decades. By 2025, CRC is projected to be the most dominant cancer in Vietnamese men and second-most dominant in women [3]. It has been reported that more than 80% of patients in Vietnam are diagnosed in the advanced stages (stage III or stage IV) [4]. The treatment in Asia, particularly in Vietnam, has modestly improved disease outcomes and prolonged survival in advanced and metastatic disease patients. Nevertheless, these advancements have been accompanied by markedly increased treatment costs. Cancer patients are amongst the most sensitive groups in terms of economic hardship; they are vulnerable to financial catastrophe and poverty (falling below the national poverty line) [5]. Sixty-eight percent of cancer patients in Vietnam experience financial hardship, which is higher than its Southeast Asian Nations (Thailand, Cambodia, Lao, Malaysia) [4,6]. The growing number of patients with CRC can be a large cost burden not only for an individual patient and his/her family but for society as a whole.

### New Contribution

A cost of illness analysis provides information on the economic burden of disease, which offers a sense of the magnitude of a problem. This can inform priority setting for developing health programs. In addition, increased attention is being paid to financial protection for health services users and equitable distribution of health services, and these have become top priorities of Vietnam’s universal health coverage. Still, health expenditures and out-of-pocket payments keep rising, placing an increased burden on patients and households in Vietnam [7,8]. Although there is an increasing trend in CRC incidence, no studies investigated the economic burden of CRC in Vietnam. This study aims to estimate the cost of CRC in Vietnam using a prevalence-based approach with the latest data available.

## 2. Materials and Methods

### 2.1. Study Design and Setting

The total economic cost is comprised of direct and indirect costs, which is analyzed from a social perspective. In this study, the direct cost includes direct medical costs and non-medical costs; the indirect cost is comprised of productivity loss due to morbidity and future income loss due to premature death. 

We estimated the average cost within one year of treatment using the cost of illness approach with data collected from a tertiary hospital. We then used the prevalence-based approach to determine the total cost in 2018, including both direct and indirect costs. Data were analyzed from a social perspective, where all costs are included. 

The formula for estimating the cost of CRC is as follows:

Total costs = Direct costs + Indirect costs(1)

#### 2.1.1. Study Design

##### Direct Cost

Data for the direct cost (medical cost and non-medical cost) was collected at the Hue Central Hospital (HCH). Hue central hospital is a tertiary hospital, designated as a referral-level hospital in the central region of Vietnam, which provides services to 15 million people in 14 area provinces. The oncology center, as a part of HCH, is also responsible for providing cancer-specific treatment services and technical support for other hospitals in the region [9].

**Figure 1:**
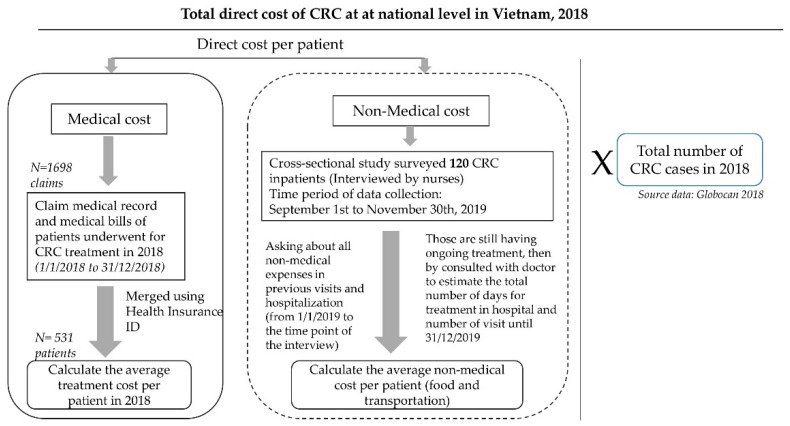
Study diagram of estimation for the direct cost of colorectal cancer in Vietnam, 2018.

As secondary data, 2018 medical records (claims data) from HCH were used to estimate the average cost of treatment per CRC patient. In addition, a hospital-based survey was also carried out to obtain the cost incurred by each patient during treatment (i.e., food and transportation expense). The data on CRC prevalence in Vietnam in 2018, by sex and age group, were retrieved from the Global Cancer Observatory, Vietnam. Population (GLOBOCAN) [10]. The average direct medical cost and direct non-medical cost was estimated per patient. This average was then used to calculate the total direct cost of CRC in Vietnam by multiplying it by the number of CRC cases in 2018 according to the GLOBOCAN [10]. A flow diagram of the estimation for direct cost is shown in Figure 1.

The formula for estimating the direct cost of CRC is as follows:

Direct costs = direct medical costs + direct non-medical costs(2)

Direct Medical Cost. First, we retrieved the treatment cost in 2018 based on medical claims via the hospital’s electronic system. To identify those was diagnosed with cancer in the year of study in medical records and electronic system, we used the diagnosis codes from the International Classification of Diseases, Ninth Revision, Clinical Modification (ICD-9-CM): C18 (colon), C19 (rectosigmoid junction), C20 (rectum). We selected all patients with CRC, either primary diagnosis or secondary diagnosis, and out of 1698 patients’ medical information hospitalizations among patients treated in 2018 at HCH, which was identified using the ICD code (C18-C20). A total of 531 CRC patients received treatment at the HCH from 1/1/2018 to 31/12/2018. The characteristics of the patients are presented in Supplementary Tables S1 and S2.

The formula for estimating the direct medical cost of CRC is as follows:

Direct medical costs = average medical costs per patient × estimated number of patients with CRC in Vietnam

Direct non-medical Cost. We conducted a survey of inpatients currently receiving treatment at the HCH to determine the direct non-medical costs. A structured questionnaire and face-to-face interviews were administered by nurses. To quantify the total daily spending at the hospital in 2019, we asked patients about their total days of hospitalization in previous visits (accumulated number of days counted from 1 January 2019 to 31 December 2019). The quantification was accomplished by acquiring the transportation and food costs of patients and caregivers. Patients were asked about their one-way transportation fees to determine the per patient transportation cost; this was then used together with the frequency of visits to determine the transportation costs for the patients and their caregivers within one year. The total food expense is defined as the length of stay multiplied by the expenditure for food per day. Spending on food was obtained by patients and caregivers separately (Food for patients and caregivers is paid separately in hopitalised bills). The characteristics of the survey respondents are presented in Supplementary Table S3. Data collection was undertaken from 1 September 2019 to 30 November 2019.

The formula for estimating the direct non-medical cost of CRC is as follows:

Direct non-medical costs = {average transportation costs per patient + average food expense per patient} * estimated number of patients with CRC in Vietnam

Estimated number patients with CRC nationwide in Vietnam in 2018. In the GLOBOCAN, the case prevalence in Vietnam in 2018 is estimated based on incident cases [11]. The case prevalence data are sorted by sex and age-group [10].

##### Indirect Cost

In this study, indirect costs consist of future income loss and productivity loss during inpatient/outpatient visits.

Future income loss: We applied a human capital approach to identify future income loss, considering the present value of the future earnings that each prematurely deceased person might have gained during his/her lifetime if the patient had lived to the average life expectancy. In particular, the human capital approach was made under the assumption that future incomes act as a substitute for future productivity, though in several cases this is not a precise representation [12]. Productivity loss due to premature death was classified as lost potential future incomes. The cost estimation equation is listed below. It includes employment rate and annual average wage by sex, according to the 2018 report from the Vietnam National General Statistics Office and the annual labor report [13,14], with a discount rate of 3% per year. Life expectancy in Vietnam is 72 years for males and 81 years for females [15].

Formula:

$$\mathrm{FIL}\;=\;\sum_{i}\sum_{j}\sum_{t}\sum_{k=1}^{n}\left\{D_{ijt}\;\times \;\left(\frac{YW_{ij\left(t+k\right)}\;\times \;E_{ij\left(t+k\right)}}{{\left(1+r\right)}^{k}}\right)\right\},$$

where

FIL = future income loss;
*i* = sex;
*j* = age;
*t* = age at death;
*k* = 1, 2,…., *n* (*k* is the difference between the age at death and the life expectancy of the age cohort);
*D* = number of deaths;
*YW* = yearly wage by *i* and *j*;
*E* = employment rates by *i* and *j*;
*r* = discount rate.

Estimated mortality from CRC in 2018: Because of a lack of reported data for mortality nationally, data on deaths were gathered from the GLOBOCAN. They computed the number of deaths based on incidence-to-mortality ratios and derived the assumption model data from cancer registries in a neighboring country (Thailand) [11]. Data on death cases were obtained for each sex and age-group [10].

Productivity loss. To calculate the loss of productivity, the total number of patient days of admission and visits were collected from the health insurance system’s medical claims data, which were then combined with age and sex-specific employment rates and monthly salaries. Age and sex-specific employment rate information was referenced from the annual labor survey 2018 [13,14].

Formula:

$$\mathrm{PL}\;=\;\sum_{i}\sum_{j}\sum_{y}\left\{\left(IV_{ij}\right)\times \;E_{ij}\;\times \;DW_{ij}\right\},$$

where

PL = productivity loss;
*i* = sex;
*j* = age;
*IV* = number of patient visits (combination of inpatient and outpatient visits);
*E* = employment rates;
*DW* = daily wage.

### 2.2. Data Analysis

A descriptive analysis was carried out to explore the distribution of characteristics of participants, their clinical status, and cost components. Frequency along with percentage and summary statistics, including mean, SD, median, and IQR (interquartile range) were used to summarize categorical and continuous variables, respectively. The statistical differences regarding the sociodemographic characteristics and clinical characteristics were examined using the Kruskal–Wallis test and Wilcoxon rank-sum (Mann–Whitney) test.

Annual discount rates of 0%, 3%, and 5% for productivity loss due to premature death were performed in the one-way sensitivity analysis [16]. Costs are presented in VND and US dollars using the 2018 exchange rate (US $1 = VND 22,880) from the State Bank of Vietnam. All data were analyzed using MS Excel and SAS version 9.1.

### 2.3. Ethical Considerations

The protocol of the study was approved by Da Nang University of Medical Technology and Pharmacy, Vietnam (Code: 0259/QĐ-HĐĐNCYSH), and agreed to by the Board of Directors of HCH, Hue city, Vietnam. All participants provided written informed consent.

## 3. Results

Table 1 presents the average direct medical cost per CRC patient collected from the HCH and the estimated total medical cost of CRC in Vietnam. The average cost per patient was VND 44 million (~$2000); the average cost for males was VND 47 million (~$2100), and the average cost for females was VND 39 million (~$1800). More expenditure for health care was noted for males. Costs were also higher for advanced stage cancer and for younger patients, which showed statistically significant differences (*p*-value < 0.001). The direct medical cost of CRC in Vietnam was estimated at VND 410,313 million (~$17.933 million); the cost was higher for males—at 225,812 million (~$9.87 million)—than for females—VND 184,500.7 million (~$8.063 million) (Table 1). The general and clinical characteristics of CRC patients in this study are shown in Supplementary Table S1 and Supplementary Table S2. The medical cost components among the 531 patients in the HCH are described in Supplementary Figure S1.

Table 2 presents the direct non-medical cost of CRC (i.e., cost of transportation and food expense). The average non-medical cost per patient was VND 9,401,000 (~$411): the travel cost was VND 812,000; cost of food for patients, VND 5,334,000; and cost of food for caregivers, VND 3,256,000. The total non-medical cost for CRC in Vietnam was estimated at VND 89,201 million (~$3.89 million). 

The general and clinical characteristics of respondents who reported their direct non-medical costs are shown in Supplementary Table S3.

Table 3 shows the estimated indirect cost of CRC. The total indirect cost was VND 2,542,369 million ($111 million). The cost related to productivity loss due to treatment was VND 29,604.11 million (~$1.294 million), and the future income loss due to premature death was VND 2,512,765 million (~$109.82 million). These cost components were highest for the 40–49 and 50–59 age groups. The indirect cost for males was relatively higher than that for females.

Table 4 provides the overall economic burden of CRC in Vietnam. The total cost of CRC was estimated at VND 3041.88 billion (~$132.9 million). The direct cost was VND 499.5 million (~21.8 million), which accounted for 16.42% of the total cost, whereas the indirect cost was VND 2542.3 billion (~$111.12 million), which is approximately 83.58 % of the total cost. The results of Table 4 are visualized in Supplementary Figure S2.

Table 5 gives the results of the sensitivity analysis with discount rates of 0% and 5% used in estimating future income loss. The cost of future income loss increased by a large percentage when the discount rate was set at 0% (total cost: $263.1 million) and declined when the discount rate was set at 5% (total cost: $92.18 million).

## 4. Discussion

This study found that the economic burden of CRC in Vietnam is substantial (VND 3041.88 billion (~$132.9 million)); it accounted for approximately 0.055% of the 2018 gross domestic product [17]. Direct costs made up 16.42% of the total economic burden, while indirect costs accounted for 83.58%. The direct medical costs related to CRC were about 0.10% of all health expenditures in 2018 [16]. Younger patients and those in advanced stages of disease bear the largest economic burden, including hospital fees and other costs incurred during hospitalization (medical costs: VND 44 million, ~$1900; non-medical costs: VND 9.4 million, ~$411). Regarding the indirect cost, 82.61% of the total cost was due to future income loss.

These findings provide information on the total economic cost of CRC in Vietnam as well as its cost components. There has been limited research estimating similar economic burdens in Vietnam. However, a large-scale study on the economic cost of tobacco-related diseases in Vietnam shows a higher cost than found in this study. It estimated that the total economic cost of smoking-attributable diseases was US$1173.2 million in 2013 [18].

Notably, medical costs made up the majority of expenses for patients, accounting for 82.1% of their direct costs. These results are consistent with previous findings in other Asian countries. A study by Vahdatimanesh and colleagues showed that direct medical costs accounted for 76.5% of direct costs in Iran (total cost: $298.15 million) [19], and a study in South Korea found that direct medical costs accounted for over 80% of direct costs in 2010 (total cost: 3.1 trillion KRW) [20]. The variation in results, in terms of either direct cost or total economic burden, between studies can be partially explained by the methodology used and the health care system/policy of each country [21]. As these studies employ diverse approaches, the direct and indirect cost proportions are likewise reported differently. In Iran, researchers found that the direct and indirect costs respectively accounted for 58% and 42% of the total burden [19], whereas in Korea, these costs were 62.82% and 37.1%, respectively [20].

In terms of the medical cost per CRC patient, our findings were consistent with the findings of a previous study conducted in Ho Chi Minh city, the largest city in Vietnam, which estimated the cost to be $2741 [22]. The average medical cost for CRC treatment is similar to other developing countries in Asia, such as Malaysia, China, and Iran [19,23,24], and the cost proportion components showed a similar distribution [19,25]. Comparing the medical cost with that for other types of cancer, the CRC finding is in line with the results of the 2014 national survey for the three most common cancers (at respiratory, at digestive, and at reproductive system), which found that the 12-month follow-up cost was 43.818 million [6], and the CRC cost was almost double the cost of breast cancer ($975 per patient in 2013) [26] or cervical cancer ($368–$11,400) [27].

Under the universal health coverage policy, 87% of the Vietnamese population is covered by social health insurance. Poor households are partially subsidized [28]. This finding is consistent with ours; we found that 92% of patients have social health insurance, which covers 80% to 100% of services, depending on the target procedures, as regulated by health insurance. However, burden is still high despite the cost of the majority of services (check-ups, tests, and treatment) were covered by health insurance for the most patients, but the patients also need to pay the remaining costs out of pocket (drugs after discharge and non-medical costs) [29]. Based on the ACTION study, a survey on the cost incurred for surgical cancer care in eight Southeast Asian countries, the number of patients in Vietnam that experience financial catastrophe after one year following a cancer diagnosis appears to be the highest in South-east Asia (68%), even though the death rate (25% of all cancer patients) is similar to Thailand and lower than other countries [30]. In addition, the ACTION study reveals that 60% of individuals with surgically operable cancer experience economic hardship in the low-middle income countries (including Vietnam, Indonesia, Laos, and the Philippines). Moreover, economic hardship was associated with a 48% rise in the risk of treatment discontinuation (no hospitalization) [31]. Additionally, we found that the non-medical expenses during hospitalization for CRC were substantial, reaching 17.8% of the direct cost. This is relatively higher than that found in a study on the Chinese population (8.3%) [24].

Our study showed that indirect costs account for 83.58% of the total cost. One explanation for this is that CRC occurs in productive age groups (15–54 years, which accounts for 58% of the Vietnamese population). In Vietnam, most people work until their late 50 s (unemployment rate, 2.0%; average retirement age for men is 60 years and for women, it is 55 years) [13,14]. Further, CRC that appears at earlier ages or is diagnosed at advanced stages creates a huge loss in terms of future income because the life expectancy for Vietnamese people is 72 years for males and 81 years for females [15].

Regarding the method for indirect cost estimation, our approach was similar to a previous study in South Korea in 2019. That study found that the indirect cost was approximately 77.91% of the total cost, and future income lost accounted for the largest percentage of the cost components [32]. In Iran, researchers found that it accounted for 55% of the total cost (not including productivity loss or job loss) [19]. The difference could be explained by the differences in the cost estimation methods and cost components used in previous studies, which greatly limits comparisons across countries. In our results, overhead costs were not included since the data are not available, which results in a low proportion of the direct costs and a high proportion of indirect costs. Further, a various methodology is used in the economic cost estimation, which makes direct international and national comparisons difficult [12]. Although studies have reached different results, it is more important to observe whether there is consistency in terms of conclusion and direction. Much work remains to determine the overall economic burden.

The rapid increase in the incidence of CRC within the last decade has led to increased expenditures, even though we have seen great improvement in treatment and survival. The largest cost is associated with late-stage CRC (III, IV) rather than early stage CRC (I and II) as late-stage CRC is potentially linked to longer hospitalization and more expensive advanced therapies. For example, 62.7% of patients diagnosed with advanced stage CRC (III, IV) in our research contributed towards the high medical expenses in total medical cost. This finding is consistent with previous studies on Vietnamese cancer patients [5,31] and those in other countries [21,24,33]. Our findings showed that the high-cost burden, on average, was borne by the younger age groups, which can be explained by the early age onset of CRC. A recent study found that the average age of initial CRC diagnosis is ten years younger in Asian countries than in other regions [22]. A confirmed trend has been noted in other populations (Korea, Taiwan, Japan, and Hong Kong) [34]. This phenomenon is also found in Vietnamese CRC patients. The proportion of early-onset (younger than 50 years) CRC cases was recorded as 28% [35]. Empirical evidence shows the early diagnosis of cancer is effective for successful treatment, decreased mortality, extended survival, and cost savings compared to late diagnosis [36]. These findings suggest the possibility that early detection and treatment are cost-effective. Vietnam, like other low-middle income countries, is facing financial constraints in implementing cancer screening programs nationally, although CRC could be a candidate for effective screening. For example, it is estimated that the cost for CRC treatment in the New Zealander population would sharply increase from $83.6 million (2014) to $100.2 million (2026), if no systematic screening program is introduced [37].

This study illustrates that the economic burden of CRC in Vietnam is significant, and this cancer imposes enormous costs on patients, their families, and society. These findings can inform budget allocation decisions for the national healthcare system, and the range of each cost component should also be understood, including age and cancer stage. Therefore, the findings in this study provide valuable information on the magnitude of CRC and the need to prioritize cancer control in Vietnam. Further studies, such as an economic evaluation of CRC screening programs, are needed. The result of this research provides insights on the cost burden of CRC, which could have significant implications for policymakers and health insurance in assisting patients most in need of financial support.

### Limitation

Some limitations should be addressed. First, the data on direct medical and non-medical costs were collected from a single hospital and may not be generalized. Interpretation of the results should be causative, especially the estimated non-medical cost, which consisted of a small sample size (120 patients) that is decreasingly representative of the entire population in Vietnam. However, the HCH, a designated referral hospital in the central region of Vietnam, is responsible for providing cancer care in an area that includes 14 provinces and has a population of 15 million. Second, the economic burden in this study may be underestimated because the costs of additional drugs after discharge and hospital overhead costs were not included in the direct medical cost. Moreover, the indirect cost of job loss due to cancer care was not included. Therefore, the cost of CRC in this study is a conservative estimation. Third, the estimated number of CRC deaths in this study may differ from the actual number of CRC deaths in Vietnam. Due to lack of data, it was estimated based on incidence-to-mortality ratios provided by GLOBOCAN and not based on cause of death statistics. Fouth, it is noted that the estimation of indirect costs based on market productivity raises concerns in countries with high levels of unemployment. Despite these limitations, the study certainly adds to our understanding of the economic burden of CRC in Vietnam. The estimation of direct cost used data from a tertiary public hospital system, which is highly representative because the treatment regimen and facilities allocation are standardized across Vietnam. Moreover, this is the first study to estimate the economic cost of CRC in Vietnam.

## 5. Conclusions

The economic burden of CRC at the national level is substantial, which should be taken into consideration in policymaking to control cancer. The medical cost for CRC diagnosis and treatment is higher in patients with advanced stage cancer and in those diagnosed at a younger age. Indirect costs account for most of the total cost because CRC occurs in the most productive age groups. The government should prioritize extending social health insurance to offset and subsidize the out-of-pocket costs for all cancer patients. Strategies to decrease the economic burden of CRC at the patient level and national level, such as screening programs, should be developed and implemented in Vietnam.

## Figures and Tables

**Table 1:** Average Medical Cost of Colorectal Cancer per Patient and Estimated Total Medical Cost at National Level in Vietnam, 2018.

	Average Medical Cost per Patient (1000 VND, Estimated from 531 Patients, 1698 Claims)				Number of CRC Patient in Vietnam (GLOBOCAN)	Total Medical Cost	
Variables	C18	C20	C19	All		Million VND	USD ($ 1)
**All (n)**	268	256	7	531			
Total cost *	41,008	47,594	30,656	43,277	9481	410,313,103	17,933,265
Insurer (96.8%)*	37,673	44,308	29,050	40,758			
**Gender** *							
Male	42,687	52,396	30,826	46,492	4857	225,812,313	9,869,419
Female	38,740	40,461	30,528	39,900	4624	184,500,789	8,063,845
**Age** * (%)							
<30 (3.2)	41,211	71,868	NA	50,228	184	9,859,937	430,941
30–39 (7.7)	56,404	55,146	NA	55,852	364	20,947,267	915,527
40–49 (12.1)	52,586	51,009	NA	51,822	1235	64,590,778	2,823,024
50–59 (27.9)	44,534	52,835	10,315	48,615	2529	122,163,179	5,339,300
60–69 (26.0)	38,304	49,544	28,166	42,881	2644	115,622,800	5,053,444
70+ (23.2)	28,723	34,308	NA	31,338	2525	77,129,143	3,371,029
Average cost (CRC stages)*							
I (6.6%)	19,696	19,406	246,300	18,974			
II (23.2%)	37,687	43,033	69,066	40,767			
III (33.9%)	48,507	52,308	13,775	49,934			
IV (28.8%)	47,433	62,704	51,978	53,581			
NA (7.5%)	12,794	13,645	NA	13,113			

Colon (C18); Rectum (C20); Rectosigmoid junction (C19); *: statistical significance (*p* < 0.05); Exchange rate in 2018: US $1 = VND 22,880. VND: Vietnam Dong.

**Table 2:** Average Non-Medical Cost of Colorectal Cancer per Patient and Estimated Total Non-Medical Cost at National Level in Vietnam, 2018 (1000 VND).

	Average Cost—Estimated from 531 Patients					Number of CRC Patient in Vietnam (GLOBOCAN)	Total Non-Medical Cost	
Cost by Gender	No of patients	Transportation	Food-Patients	Food-Caregiver	Cost per Patient VND (USD)		VND (1000)	US ($ 1)
All	120	487	5334	3256	9561 (411)	9481	89,201,728	3,898,677
Male	67	302	5422	3302	9328 (407)	4857	45,306,096	1,980,162
Female	53	537	5223	3197	9493 (415)	4624	43,895,632	1,918,515

Exchange rate in 2018: US $1 = VND 22,880. VND: Vietnam Dong.

**Table 3:** Estimated Total Indirect Cost for CRC at National Level in Vietnam, 2018 (VND million).

Gender	Age Groups					Number of CRC in Vietnam (GLOBOCAN)	Total indirect Cost	
	<30	30–39	40–49	50–59	60+		Total(VND)	US $
**Productivity loss due to cancer care**						Prevalent cases		
Male	619.5	1854.5	7777.7	8328.1		2328	18,579.8	812,056
Female	770.4	1469.7	3226.3	5557.9		1985	11,024.3	481,830
All	1389.8	3324.3	11,004.0	13,885.9		4313	29,604.1	1,293,886
**Future income loss due to premature deaths**						Death cases		
Male	11,649.4	109,365.7	369,649.9	611,940.3	276,625.1	2492	1,379,230.5	60,281,052
Female	8236.7	82,466.9	246,792.9	405,467.2	390,571.1	2438	1,133,534.9	49,542,611
All	19,886.2	191,832.7	616,442.8	1,017,407.5	667,196.2	4930	2,512,765.4	109,823,664
**The total of indirect cost**								
Male	12,268.9	111,220.4	377,427.6	620,268.4	276,625.1		1,397,810.3	61,093,109
Female	9007.1	83,936.7	250,019.2	411,025.0	390,571.1		1,144,559.2	50,024,441
All	21,276.1	195,157.0	627,446.8	1,031,293.5	667,196.2		2,542,369.5	111,117,550

Exchange rate in 2018: US $1 = VND 22,880. VND: Vietnam Dong.

**Table 4:** Estimated total economic burden of CRC at national level in Vietnam, 2018 (VND million).

Cost Components	Male	Female	All (VND)	US $	Percentage
**Direct cost**	271,118.4	228,396.4	499,514.8	21,831,942	16.42%
Medical cost	225,812.3	184,500.7	410,313.1	17,933,265	13.49%
Non-medical cost	45,306.1	43,895.6	89,201.7	3,898,677	2.93%
**Indirect cost**	1,397,810.3	1,144,559.2	2,542,369.5	111,117,550	83.58%
Future income loss	1,379,230.4	1,133,534.9	2,512,765.4	109,823,664	82.61%
Productivity loss	18,579.8	11,024.2	29,604.1	1,293,886	0.97%
**Total**	1,668,928.7	1,372,955.6	3,041,884.4	132,949,492	0.055%~ GDP
$ US	72,942,689.37	60,006,802.32			

Exchange rate in 2018: US $1 = VND 22,880. GDP: Gross domestic product. VND: Vietnam Dong.

**Table 5:** One-way sensitivity analysis of cost of colorectal cancer, applying the discount rate 0% and 5%.

Costs	0%	3% (base)	5%
Indirect cost (million VND)	5,520,336.9	2,542,369.5	1,609,748.3
Total cost (million VND)	6,019,851.7	3,041,884.3	2,109,263.1
**USD**	263,105,409	132,949,492	92,188,075

Exchange rate in 2018: US $1 = VND 22,880. VND: Vietnam Dong.

## Supplementary Materials

The following are available online at https://www.mdpi.com/1660-4601/18/1/12/s1, Figure S1: Percentage of medical cost components for CRC patients, Hue Central Hospital, Vietnam 2018 (n = 531), Figure S2: Direct cost and indirect cost of CRC at national level in Vietnam, 2018, Table S1: Number of CRC cases for each data source in estimation of total cost at national level in Vietnam, 2018, Table S2: Characteristics of patients with colorectal cancer and their clinical characteristics used in estimation of direct medical cost, Hue Central Hospital, 2018, Table S3: Characteristics of respondents who provide data on direct non-medical cost, Hue Central Hospital, 2019 (n = 120).
