# Supplementary material for: The Direct and Indirect Costs of Colorectal Cancer in Vietnam: An Economic Analysis from a Social Perspective
Source: Int J Environ Res Public Health. 2020 Dec 22;18(1):12. doi: 10.3390/ijerph18010012 (PMC7792935; doi:10.3390/ijerph18010012)
Supplement: Supplementary file 1 [file ijerph-18-00012-s001.pdf]

Supplementary Materials

Supplementary Table S1. **Number of CRC cases for each data source in estimation of total cost at national level in Vietnam, 2018**

Variables	Original studies (to estimate average cost per patient)				Number of CRC in Vietnam (expected cases to estimate the total economic burden)					
	**Medical claims 2018**		**Hospital-based survey**		**GLOBOCAN (2018)**					
	N=531		N=120*		*Prevalent cases*			*Death cases*		
	Male	Female	Male	Female	All	Male	Female	All	Male	Female
All	311 (58.6)	220 (41.4)	67 (55.8)	53 (44.2)	9,481 (100.0)	4,857 (51.2)	4,624 (48.8)	8,104 (100)	4,116 (50.8)	3,988 (49.2)
Age (mean±SD)	55.86 ± 15.6 (min,max: 1-98)		57.2 ± 13.2 (min, max: 20-80)							
Less than 30	11 (3.5)	6 (2.7)	2 (3.0)**	2 (3.8)**	184 (1.9)	77 (1.6)	107 (2.3)	67 (0.8)	33 (0.8)	34 (0.9)
30-39	18 (5.8)	23 (10.5)	5 (7.5)	5 (9.4)	364 (3.8)	177 (3.6)	187 (4.0)	249 (3.1)	128 (3.1)	121 (3.0)
40-49	34 (10.9)	30 (13.6)	7 (10.4)	5 (9.4)	1,235 (13.0)	694 (14.3)	541 (11.7)	786 (9.7)	435 (10.6)	351 (8.8)
50-59	100 (32.2)	49 (22.3)	16 (23.9)	14 (26.4)	2,529 (26.7)	1,357 (27.9)	1,172 (25.3)	1,592 (19.6)	880 (24.4)	712 (17.9)
60-69	89 (28.6)	47 (21.4)	26 (38.8)	20 (37.7)	2,644 (27.9)	1,389 (28.6)	1,255 (27.1)	1,886 (23.3)	1,016 (24.7)	870 (21.8)
70+	59 (19.0)	65 (29.5)	11 (16.4)	7 (13.2)	2,525 (26.6)	1,163 (23.9)	1,362 (29.5)	3,524 (43.5)	1,624 (39.5)	1,900 (47.6)

*: study surveyed those ≥20. ; **: those aged 20-29 years; data present as number and percentage. Age groups: Percentage was presented in parenthesis.

Supplementary Table S1. ***Characteristics of patients with colorectal cancer and their clinical characteristics used in estimation of direct medical cost, Hue Central Hospital, 2018***

**Variables**	**Type of cancers**								
	**Colon (C18)**		**Rectum (C20)**		**Rectosigmoid junction (C19)**		**All**		*p-value**
	n	%	n	%	n	%	n	%	
All	268	50.5	256	48.2	7	1.3	531	1	
Gender									0.60
Male	154	57.5	153	59.8	3	42.9	310	58.4	
Female	114	42.5	103	40.2	4	57.1	221	41.6	
Age									0.020
Less than 30 years	12	4.5	5	2.0	0	0.0	17	3.2	
30-39	23	8.6	18	7.0	0	0.0	41	7.7	
40-49	33	12.3	31	12.1	0	0.0	64	12.1	
50-59	65	24.3	81	31.6	2	28.6	148	27.9	
60-69	78	29.1	58	22.7	2	28.6	138	26.0	
70-79	42	15.7	37	14.5	0	0.0	79	14.9	
80+	15	5.6	26	10.2	3	42.9	44	8.3	
Health Insurance									0.33
0	6	2.2	10	3.9	1	14.3	17	3.2	
less than 50%	9	3.4	4	1.6	0	0.0	13	2.4	
80%	93	34.7	74	28.9	2	28.6	169	31.8	
95%	22	8.2	18	7.0	0	0.0	40	7.5	
100%	138	51.5	50	19.5	4	57.1	192	36.2	
Stages									0.15
CRC stage I	14	5.2	20	7.8	1	14.3	35	6.6	
CRC stage II	57	21.3	65	25.4	1	14.3	123	23.2	
CRC stage III	82	30.6	95	37.1	3	42.9	180	33.9	
CRC stage IV	90	33.6	61	23.8	2	28.6	153	28.8	
NA	25	9.3	15	5.9	0	0.0	40	7.5	
Frequency of hospitalization									0.059
1 time	142	53.0	132	51.6	7	100.0	281	52.9	
<=5 times	67	25.0	84	32.8	0	0.0	151	28.4	
>5 times	59	22.0	40	15.6	0	0.0	99	18.6	
Mean (times)	3.4		**3.1**		1		3.2		
Days of hospitalization									
Per visit (mean/SD)	10.9 (9.36)		15.4 (15.9)		17.4 (9.6)		13.1 (12.9)		
No. day within a year (mean)	36.96269		47.09766		24.85714		41.68927		

p-value: chi-square test*

Supplementary Table S3. ***Characteristics of respondents who provide data on direct non-medical cost, Hue Central Hospital, 2019 (n=120)***

**Variables**	**Number (n)**	**Percent (%)**
Age group		
20-39	14	11.67
40-59	42	35
60+	64	53.33
Age - Mean/SD, (min-max)	57.2/13.23	(20-85)
Gender		
Male	67	55.83
Female	53	44.17
Jobs		
Business	6	5

Variables	Number (n)	Percent (%)
Employment	19	15.83
Agriculture	51	42.5
No job/older	44	36.67
Region		
Urban	38	31.93
Rural	82	68.07
Economic		
Poorest	4	3.36
Near poor	33	27.73
Fair	82	68.91
Marital status		
married	95	79.17
Divorce	5	4.17
Single	20	16.67
Education		
primary	52	43.33
Secondary	35	29.17
High school	14	11.67
College	19	15.83
Health insurance		
Yes	112	93.33
95%	57	47.5
100%	55	45.83
No	8	6.67
Types of cancer		
C18	44	36.67
C20	76	63.33
CRC Stages		
I	4	3.33
II	24	20
III	64	52.5
IV	28	24.1

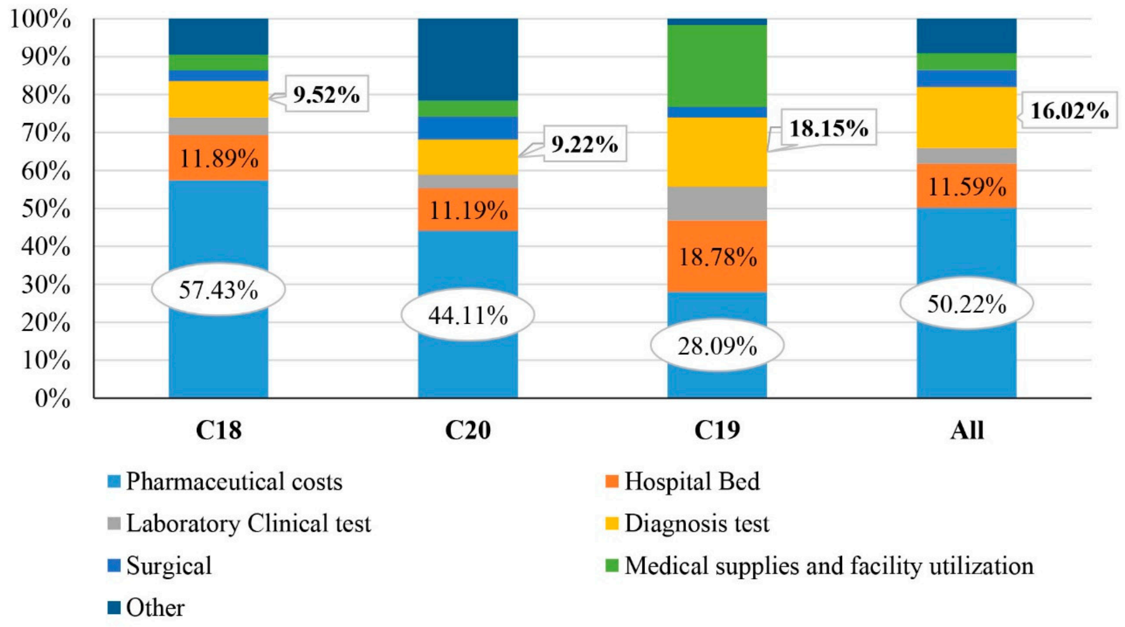

Supplementary Figure S1. Percentage of medical cost components for CRC patients, Hue Central Hospital, Vietnam 2018 (n=531)

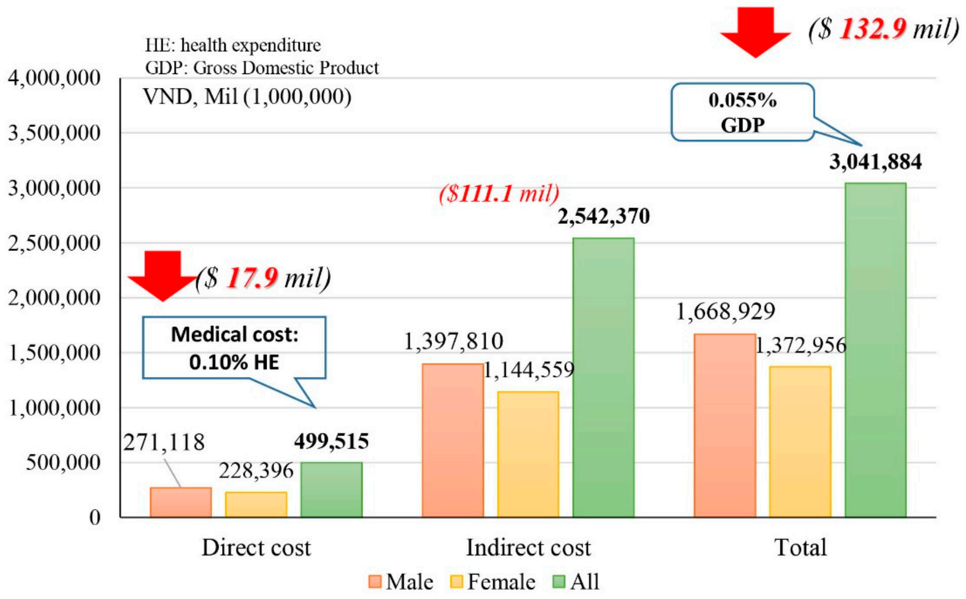

Supplementary Figure S2. Direct cost and indirect cost of CRC at national level in Vietnam, 2018 *(Mil, Million), Exchange rate in 2018: US $1 = VND 22,880*
